# Does place of residence affect risk of suicide? a spatial epidemiologic investigation in Kentucky from 1999 to 2008

**DOI:** 10.1186/1471-2458-12-108

**Published:** 2012-02-08

**Authors:** Daniel M Saman, Sabrina Walsh, Anna Borówko, Agricola Odoi

**Affiliations:** 1 Department of Epidemiology, College of Public Health, University of Kentucky, Lexington, Kentucky, USA; 2 Department of Health Services Management, College of Public Health, University of Kentucky, Lexington, Kentucky, USA; 3 Department of Biomedical & Diagnostic Sciences, College of Veterinary Medicine, University of Tennessee, Knoxville, Tennessee, USA

## Abstract

**Background:**

Approximately 32,000 people take their own lives every year in the United States. In Kentucky, suicide mortality rates have been steadily increasing since 1999. Few studies in the United States have assessed spatial clustering of suicides. The purpose of this study was to identify high-risk clusters of suicide at the county level in Kentucky and assess the characteristics of those suicide cases within the clusters.

**Methods:**

A spatial epidemiological study was undertaken using suicide data for the period January 1, 1999 to December 31, 2008, obtained from the Kentucky Office of Vital Statistics. Descriptive analyses using Pearson's chi-square test and t-test were performed to determine whether differences existed in age, marital status, year, season, and suicide method between males and females, and between cases inside and outside high-risk spatial clusters. Annual age-adjusted cumulative incidence rates were also calculated. Suicide incidence rates were spatially smoothed using the Spatial Empirical Bayesian technique. Kulldorff's spatial scan statistic was applied on all suicide cases at the county level to identify counties with the highest risks of suicide. Temporal cluster analysis was also performed.

**Results:**

There were a total of 5,551 suicide cases in Kentucky from 1999 to 2008, of which 5,237 (94%) were included in our analyses. The majority of suicide cases were males (82%). The average age of suicide victims was 45.4 years. Two statistically significant (p < 0.05) high-risk spatial clusters, involving 15 counties, were detected. The county level cumulative incidence rate in the most likely high-risk cluster ranged from 12.4 to 21.6 suicides per 100,000 persons. The counties inside both high-risk clusters had relative risks ranging from 1.24 to 1.38.

**Conclusions:**

Statistically significant high-risk spatial clusters of suicide were detected at the county level. This study may be useful for guiding future research and intervention efforts. Future studies will need to focus on these high-risk clusters to investigate reasons for these occurrences.

## Background

Between 2000 and 2007, there were 256,085 deaths in the United States (US) attributed to suicide. Approximately 32,000 people take their own lives every year in the US [1]. In 2009, suicide was the tenth leading cause of death for all ages, the second leading cause of death among 25-34-year olds, and the third leading cause of death among 15-24-year olds [1]. Firearms, suffocation, and poisoning are the most common methods of suicide; however, men and women differ in the methods used. In the same year, firearms were the most commonly used methods of suicide among males, while poisoning was the most commonly used mechanism in females. Males died by suicide at nearly four times the rate of females and represented 78.8% of all US suicides. During their lifetime though, women attempt suicide about two to three times as often as men [1].

In Kentucky, suicide mortality rates have been steadily increasing since 1999. In 2006, suicides rose to 14.4 per 100,000 persons from the 1999 rate of 11.3 per 100,000 persons, a 27% increase. With an average of 13.4 suicides per 100,000 people annually (2000-2006), Kentucky ranks 16^th ^highest for suicide in the US [1]. Additionally, medical costs and lost wages associated with suicide also take their toll on communities. In 2005, suicide cost society $26.7 billion in combined medical and work loss costs, while in Kentucky it was estimated to cost $481 million [2].

A combination of demographic, individual, relational, community, and societal factors contribute to the risk of suicide. According to the World Health Organization's (WHO) report on violence and health, demographic factors such as age and sex, psychiatric, biological, social and environmental factors, as well as factors related to an individual's life history might play a role in making people more likely to attempt or commit suicide [3].

Although much is understood about suicide at the individual level, including multiple factors associated with increased risk of suicide [3], little has been done at the ecologic level to identify counties or neighborhoods with the greatest risk of suicide. Just as determining individual-level risk factors for suicide is vital for suicide prevention efforts, identifying high-risk areas and investigating spatial patterns for suicide provides a richer understanding of the determinants of suicide than the individual-level risk factors alone. Thus, identifying high-risk counties using spatial statistics may allow for a better targeting of resources and suicide intervention efforts so as to prevent future suicides.

Several studies have used spatial statistical techniques in assessing the presence of high-risk clusters including a brain cancer cluster study [4], a study on networks of sexually transmitted infection [5], and on breast cancer mortality disparities [6]. Other studies incorporating similar methodologies have assessed high-risk clusters of La Crosse virus in West Virginia [7], clusters of giardiasis in Canada [8], and clustering of lung cancer in Italy [9]. However, spatial studies of suicide clusters have been limited. Exeter and Boyle (2007) found a significant geographical cluster of suicide among young adults in east Glasgow across three time periods (1980 to 1982, 1990 to 1992, and 1999 to 2001), which were attributed to socioeconomic deprivation [10]. Another study investigating suicide clusters in Queensland, Australia, found clusters in low socioeconomic areas [11]. These studies provide some support that regions at high risk for suicide are those with greater socioeconomic deprivation.

Though suicide studies have used spatial statistical techniques in other countries, little has been published in the US. The present study has the potential to bridge the gap between suicide research and targeted prevention. The primary purpose of this study was to identify counties at the highest risk for suicide. Secondarily, this study also tests whether suicides are clustered temporally. Thus, the current study provides an analytical framework, using spatial statistics, to identify and target areas with the highest risk of suicide. Further, identification of counties at a greater risk for suicide is expected to guide resources and assist in policy decision making at the county level.

## Methods

### Study design

This is an ecological study of suicides in Kentucky counties (n = 120) from January 1, 1999 to December 31, 2008. All the cases of suicide identified in the study occurred in Kentucky. This research was approved by the University of Kentucky Institutional Review Board (IRB #: 02-0441-p6h).

**Figure 1:**
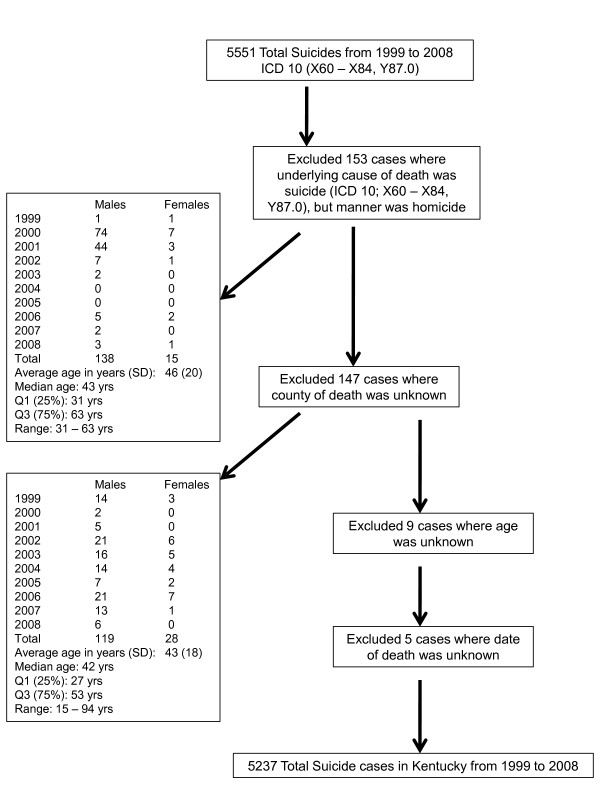
**Exclusion criteria used in selection of cases for this study**.

The Kentucky Office of Vital Statistics provided electronic injury-related death certificate data files from January 1, 1999 to December 31, 2008. A subset of the data was then generated using The *International Classification of Diseases*, 10th revision (ICD-10) External Causes of Death Codes (X60-X84, Y10-Y34, Y87.0), which meet the Center's for Disease Control and Prevention's (CDC) National Violent Death Reporting System's definition of suicide [12]. Data elements included gender, age, marital status, date of suicide, county of suicide, and suicide method. Several exclusion criteria were applied: 1) cases were first excluded if the underlying cause of death did not match the manner of death; 2) from this set, cases with unknown county of death were excluded; 3) thirdly, any cases where age was unknown were excluded; 4) lastly, cases where the date of death was unknown were also excluded (see Exclusion Criteria in Figure 1). After these cases were excluded, the remaining data set did not have any missing values.

### Descriptive statistical analyses

We used Pearson's chi-square test and two-tailed unequal variance t-tests to determine whether there were significant differences between cases by gender for age, marital status, year, season, and suicide method. Pearson's chi square tests and t-tests were calculated in WinPepi v11.4 [13] and SAS v9.3 (SAS Institute Inc, Cary, NC) [14], respectively.

Age adjusted male and female cumulative incidence rates were directly adjusted using the decennial 2000 United States Census Population. Cumulative incidence rates were expressed as the number of suicides per 100,000 persons for all of Kentucky from 1999 to 2008, and calculated in Microsoft Excel 2007 [15].

### Geographic analysis

Smoothed cumulative incidence suicide rates were mapped at the county level (n = 120). The heterogeneity of variances and spatial autocorrelation of suicide rates were adjusted for by smoothing using Spatial Empirical Bayesian (SEB) rate smoothing [16]. This technique is appropriate when population sizes of areas of aggregation vary and there is spatial autocorrelation in the data. Given that population sizes vary by county, suicide rates from counties of small population have greater variance than counties with higher populations [17]. This technique was implemented in GeoDa v0.95i [18] using a first-order queen contiguity spatial weights matrix [19].

The geographic boundary file used in this study was downloaded from the United States Census, TIGER, Geodatabase [20]. ArcGIS v10 (ESRI, Inc, Redlands, CA.) [21] was used to create the cartographic displays of smoothed incidence rates, with single hue color schemes generated by http://ColorBrewer.org[22]. The SEB smoothed incidence rates were grouped into five classes/categories using the Jenks natural breaks optimization algorithm [23].

### Spatial and temporal scan statistical cluster analyses

The detection of high-risk local spatial clusters of suicide was performed using Kulldorff's 2-dimensional spatial scan statistic [24], and implemented in SaTScan v9.1 [25]. The advantages of using the scan statistic include controlling for covariates, adjusting for multiple comparisons and various population sizes, and limiting preselection bias by not specifying *a priori *the observed set of cases within a cluster [24,26,27]. Significance testing is performed using Monte Carlo simulation where the null hypothesis of no cluster is rejected at an α level of 0.05 if the simulated p-value is less than or equal to 0.05 [28]. A significant high-risk cluster is interpreted as having an increased risk of suicide within the circular window relative to outside [27]. We also used Pearson's chi-square tests and two-tailed unequal variance t-tests to determine whether the characteristics of suicide victims that fell within the identified spatial clusters were significantly different from the state distribution. For the purely spatial analysis, a discrete Poisson probability model was used to scan for non-overlapping geographical areas (counties) with statistically significant high rates of suicide, using a maximum spatial cluster size of 50% of the total population at risk, as recommended by Kulldorff [24].

Further, high-risk temporal trends were investigated using the temporal scan statistic [25]. The temporal scan statistic uses a window that moves in one dimension, time, to identify a time period where suicide risk was higher than outside that time period. For the purely temporal analysis, we used a discrete Poisson probability model using a maximum temporal cluster size of 1-year and a 1-year time aggregation.

For statistical inference, 19,999 Monte Carlo replications were performed for all analyses, and the null hypothesis of no clusters was rejected at a probability (*p*) value of ≤ 0.05. All analyses were implemented in SatScan and adjusted for age (10-14, 15-19, 20-24,... ≥ 85) and gender distributions at the county level for each individual year from 1999 to 2008 using population estimates derived from the US Census Bureau's Population Estimates Program obtained from the Surveillance, Epidemiology, and End Results (SEER) Program at the National Cancer Institute [29]. Cartographic displays of spatial clusters were made using ArcGIS 10 [21].

## Results

### Descriptive epidemiology of suicide mortality

Of the 5,551 suicide deaths abstracted from death certificates from the Kentucky Office of Vital Statistics between 1999 and 2008, 5,237 were used in our analyses after applying our exclusion criteria (see Exclusion Criteria in Figure 1). The excluded cases where the underlying cause of death did not match the manner of death (153 cases) appeared to be differentially distributed by year, where 128 (84%) of those excluded cases occurred in 2000 and 2001. Among the other excluded cases, no apparent differential patterns were present.

Differences in age, marital status, year, seasons, and suicide method among suicide victims by gender are shown in Table 1. There were 4,313 (82%) male suicide cases. The average age of suicide victims was 45.4 years, with significant differences between males (45.6 years old) and females (44.3 years old). Among age categories, males and females also showed significant differences (*p *< 0.001). There were also significant differences by marital status, as 31% of females were divorced versus 22% of males (*p *< 0.001). Overall, 42% of all cases were married, 26% were single or never married, 24% were divorced, and 7% were widowed. Males and females differed significantly in the suicide method (*p *< 0.001), where 34.1% of female victims used self poisoning versus 7.7% of male victims. Male suicide victims used firearms with a greater frequency than females (71.1% vs 48.8%). Overall, 3,517 (67.2%) suicides were firearm related.

**Table 1:** Characteristics of suicide cases in Kentucky, 1999-2008

Variable	Male	Female	Total	Pearson × ^2 ^(df)
*Suicide (row% of total)*	4,313 (82)	924 (18)	5,237 (100)	
Average Age (SD)*	45.6 (18.0)	44.3 (15.4)	45.4 (17.6)	-2.32, *p = *0.0203 (1,533)
*Age Categories (column % of sex specific total)*				56.646, *p *= 0.001 (15)
10-14 yrs	20 (0.46)	5 (0.54)	25 (0.47)	
15-19 yrs	183 (4.2)	42 (4.5)	225 (4.3)	
20-24 yrs	334 (7.7)	45 (4.9)	379 (7.2)	
25-29 yrs	377 (8.7)	70 (7.6)	447 (8.5)	
30-34 yrs	382 (8.9)	79 (8.5)	461 (8.8)	
35-39 yrs	454 (10.5)	113 (12.2)	567 (10.8)	
40-44 yrs	484 (11.2)	137 (14.8)	621 (11.9)	
45-49 yrs	472 (10.9)	116 (12.6)	588 (11.2)	
50-54 yrs	385 (8.9)	97 (10.5)	482 (9.2)	
55-59 yrs	284 (6.6)	81 (8.8)	365 (7.0)	
60-64 yrs	212 (4.9)	46 (5.0)	258 (4.9)	
65-69 yrs	189 (4.4)	32 (3.5)	221 (4.2)	
70-74 yrs	156 (3.6)	22 (2.4)	178 (3.4)	
75-79 yrs	144 (3.3)	18 (1.9)	162 (3.1)	
80-84 yrs	138 (3.2)	14 (1.5)	152 (2.9)	
85-98 yrs	99 (2.3)	7 (0.76)	106 (2.0)	
*Marital Status (column % of sex spepific total)*				47.840, *p *= 0.001 (4)
Divorced	966 (22)	291 (31)	1,257 (24)	
Married	1,850 (43)	357 (39)	2,207 (42)	
Single or Never Married	1,180 (27)	189 (20)	1,369 (26)	
Widowed	296 (7)	80 (9)	376 (7)	
Not Classified	21 (0.5)	7 (0.8)	28 (0.5)	
*Year (column % of sex specific)*				3.668, *p *= 0.932 (9)
1999	382 (8.9)	78 (8.4)	460 (8.8)	
2000	339 (7.9)	68 (7.4)	407 (7.8)	
2001	370 (8.6)	88 (9.5)	458 (8.7)	
2002	433 (10.0)	89 (9.6)	522 (10.0)	
2003	458 (10.6)	95 (10.3)	553 (10.6)	
2004	450 (10.4)	92 (10.0)	542 (10.3)	
2005	457 (10.6)	109 (11.8)	566 (10.8)	
2006	497 (11.5)	97 (10.5)	594 (11.3)	
2007	479 (11.1)	106 (11.5)	585 (11.2)	
2008	448 (10.4)	102 (11.0)	550 (10.5)	
*Seasons (column % of sex specific total)*				0.541, *p *= 0.910 (3)
Fall	1,016 (23.6)	225 (24.3)	1,241 (23.7)	
Spring	1,145 (26.5)	236 (25.5)	1,381 (26.4)	
Summer	1,131 (26.2)	241 (26.1)	1,372 (26.2)	
Winter	1,021 (23.7)	222 (24.0)	1,243 (23.7)	
*Suicide method (Column % of sex specific total)*				492.01, *p *< 0.001 (3)
Self-poisoning (X60-X69)	333 (7.7)	315 (34.1)	648 (12.4)	
Hanging, strangulation, suffocation, drowning and submersion (X70-X71)	777 (18.0)	127 (13.7)	904 (17.3)	
Firearm (X72-X74)	3,066 (71.1)	451 (48.8)	3,517 (67.2)	
All other methods (X75-X84, Y87)	137 (3.2)	31 (3.4)	168 (3.2)	

*t-test for unequal variances used

**Figure 2:**
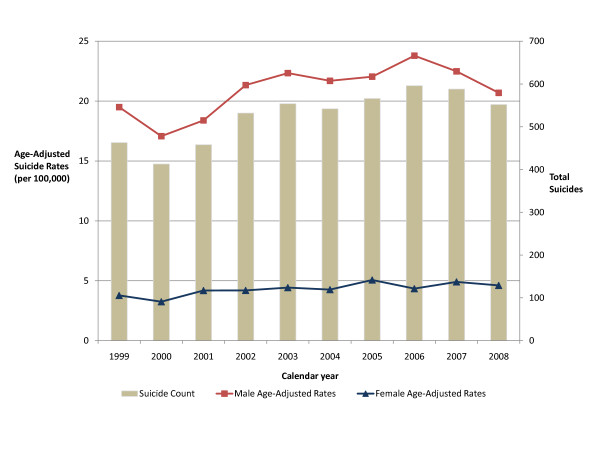
Age adjusted male and female cumulative incidence by year in Kentucky, 1999-2008*. *Average male cumulative incidence: 20.9 per 100,000 persons. Average female cumulative incidence: 4.3 per 100,000 persons.

Figure 2 displays age adjusted male and female cumulative suicide incidence rates by year along with total suicides by year. The average male cumulative incidence rate from 1999 to 2008 was 20.9 per 100,000 persons, while the average female cumulative incidence rate was 4.3 per 100,000 persons.

### Spatial distribution of suicide

**Figure 3:**
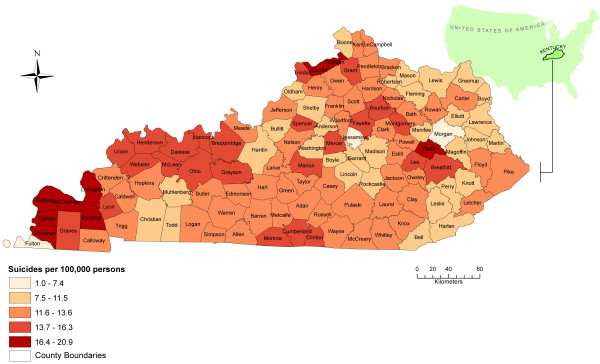
**The smoothed cumulative incidence of suicide at the county level**. The distribution of spatial empirical Bayesian (SEB) smoothed suicide incidence rates at the county level for Kentucky between 1999 and 2008.

The annual median SEB smoothed rate was 12.7 per 100,000 persons (range: 1-20.9) (Figure 3). The counties with the highest SEB suicide mortality rates (greater than 17.6 suicides per 100,000) included Carlisle (17.7 suicides per 100,000), Hickman (17.9 suicides per 100,000), Ballard (18.4 suicides per 100,000), Wolfe (20.7 suicides per 100,000), and Gallatin (20.9 suicides per 100,000) (Figure 3).

### Spatial and temporal clusters of suicide

**Figure 4:**
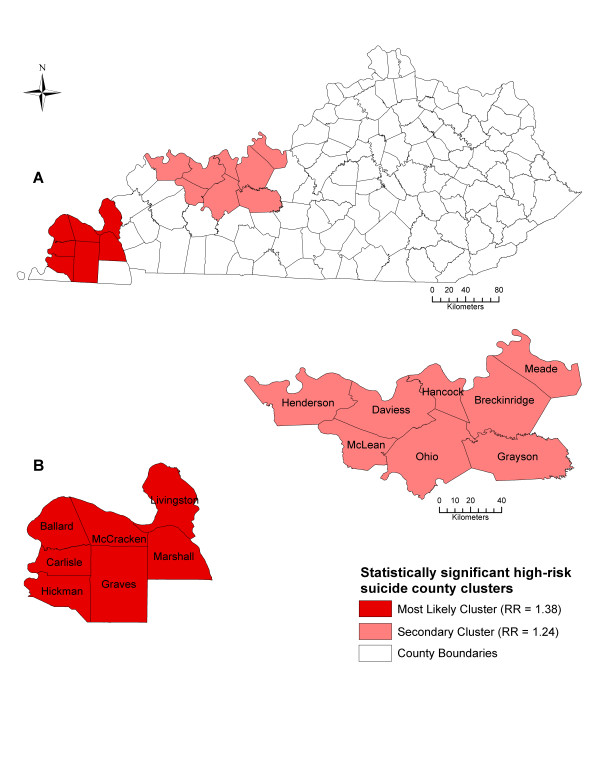
**Significant spatial clusters of high suicide mortality risks from 1999 to 2008 in Kentucky**. These maps show the statistically significant (*p *< 0.05) high-risk spatial clusters of suicide in Kentucky (**A**) at the county level (**B**) from 1999-2008 detected by Kulldorff's spatial scan statistic, controlling for age and gender. RR = relative risk.

Two significant (*p *< 0.05) high-risk spatial clusters of suicide were identified (Table 2 & Figure 4). The most likely high-risk spatial cluster of suicide comprised seven counties (Ballard, Carlisle, Graves, Hickman, Livingston, Marshall, and McCracken). Populations within the counties of the most likely cluster are at a 38% greater risk (relative risk, RR = 1.38; *p *= 0.0029) of suicide than outside the cluster (Table 2 & Figure 4). Additionally, the most likely high-risk spatial cluster had an estimated 19.8 suicides per 100,000 persons annually. A secondary high-risk spatial cluster was found among eight counties (Breckinridge, Daviess, Grayson, Hancock, Henderson, McLean, Meade, Ohio). This cluster had a 24% increased risk (RR = 1.24, *p *= 0.0324) of suicide than outside the cluster. This secondary cluster of high risk had an estimated 17.8 suicides per 100,000 persons annually (Table 2 & Figure 4).

**Table 2:** Purely spatial and temporal significant clusters of suicides in Kentucky counties, 1999-2008

Type	Counties	Observed cases	Excepted cases	Population	Annual cases/100,000 Persons	Relative risk (RR)	*p*-Value (Controlling for age and sex)	Log-likelihood ratio	Time Frame
*Purely Spatial*									
Most likely	Ballard, Carlisle, Graves, Hickman, Livingston, Marshall, McCracken	283	207.66	141,345	19.8	1.38	0.0029	12.83	1999-2008
Secondary	Breckinridge, Daviess, Grayson, Hancock, Henderson, McLean, Meade, Ohio	383	313.75	215,991	17.8	1.24	0.0324	7.62	1999-2008
*Purely Temporal*									
Most likely	All	594	535.82	-	16.1	1.12	0.04415	3.41	2006

The purely temporal cluster analysis revealed that 2006 experienced a greater than expected number of suicides, with a marginal significantly higher risk of 12% compared to the other years (RR = 1.12, *p *= 0.04415) (Table 2).

### Descriptive epidemiology of high risk spatial clusters

Table 3 compares the most likely and secondary spatial clusters to the rest of Kentucky. Only variables that had significant differences inside versus outside clusters are included in the table. The average age within the most likely spatial cluster was statistically different from the rest of Kentucky (48.9 yrs vs. 45.2 yrs) (*p *< 0.01), as was suicide cases by age categories (p = 0.02). This was also the case for the average age of males within the most likely cluster (49.3 yrs) versus outside the cluster (45.4 yrs) (*p *< 0.01). Also, 19% of suicides within the secondary spatial cluster used poisoning as the method of suicide, while only 12% used poisoning outside the cluster (*p *< 0.001). Though average age was not significantly different within versus outside the secondary cluster, suicide cases by age categories for within versus outside the cluster was significant (*p *= 0.009).

**Table 3:** Descriptive characteristics of suicide cases inside and outside both significant spatial high-risk suicide clusters

	Within Most Likely High-Risk Spatial Cluster (*column % of Suicides within cluster*)	Rest of Kentucky (*column % of Suicides outsides outside cluster*)	Pearson χ^2 ^(df)	Within Secondary High-risk Spatial Cluster (*column % of Suicides within cluster*)	Rest Kentucky (*column % of Suicides outside cluster*)	Pearson χ^2 ^(df)
*Counties*	Ballard, Carlisle, Graves, Hickman, Livingston, Marshall, McCracken	-		Breckinridge, Daviess, Grayson, Hancock, Henderson, McLean, Meade, Ohio	-	
Age*						
Average age (SD)	48.9 (18.8)	45.2 (17.5)	3.190, *p *< 0.01 (309)	45.5 (16.1)	45.4 (17.7)	-0.09, *p *= 0.932 (459)
Average Age of Males (SD)	49.3 (19.2)	45.4 (18.0)	2.92, *p *< 0.01 (250)	45.7 (16.7)	45.6 (18.2)	-0.0633, *p *= 0.950 (382)
Average Age of Females (SD)	47.1 (17)	44.1 (15.3)	-1.12, *p *= 0.267 (57)	44.3 (12.4)	44.3 (15.6)	0.023, *p *= 0.982 (75)
*Age category*						
10-14 yrs	1 (0.35)	24 (0.48)	28.264, *p *= 0.020 (15)	0 (0)	25 (0.52)	30.783, *p *= 0.009 (15)
15-19 yrs	15 (5.3)	210 (4.2)		12 (3.1)	213 (4.4)	
20-24 yrs	16 (5.6)	363 (7.3)		21 (5.5)	358 (7.4)	
25-29 yrs	13 (4.6)	434 (8.8)		30 (7.8)	417 (8.6)	
30-34 yrs	19 (6.7)	442 (8.9)		32 (8.4)	429 (8.4)	
35-39 yrs	28 (9.9)	539 (10.9)		44 (11.5)	523 (10.8)	
40-44 yrs	31 (11.0)	590 (11.9)		66 (17.2)	555 (11.4)	
45-49 yrs	29 (10.2)	559 (11.3)		37 (9.7)	551 (11.4)	
50-54 yrs	34 (12.0)	448 (9.0)		48 (12.5)	434 (8.9)	
55-59 yrs	23 (8.1)	342 (6.9)		24 (6.3)	341 (7.0)	
60-64 yrs	14 (4.9)	244 (4.9)		23 (6.0)	235 (4.8)	
65-69 yrs	13 (4.6)	208 (4.2)		11 (2.9)	210 (4.3)	
70-74 yrs	10 (3.5)	168 (3.4)		10 (2.6)	168 (3.5)	
75-79 yrs	13 (4.6)	149 (3.0)		5 (1.3)	157 (3.2)	
80-84 yrs	15 (5.3)	137 (2.8)		14 (3.7)	138 (2.8)	
85-98 yrs	9 (3.2)	97 (2.0)		6 (1.6)	100 (2.1)	
Total Suicides	283 (100)	4,954 (100)		383 (100)	4854 (100)	
*Suicide Method*						
Self-poisoning (X60-X69)	34 (12)	614 (12)	4.569, *p *= 0.206 (3)	74 (19)	574 (12)	21.027, *p *< 0.001 (3)
Hanging, strangulation, suffocation, drowning and submersion (X70-X71)	43 (15)	861 (17)		55 (14)	849 (17)	
Firearm (X72-X74)	202 (71)	3,315 (67)		247 (64)	3,270 (67)	
All other methods (X75-X84, Y87)	4 (1)	164 (3)		7 (2)	161 (3)	
Total Suicides	283 (100)	4,954 (100)		383 (100)	4,854 (100)	
*Cumulative incidence rates per 100,000 persons*						
Average (SD)	17.6 (2.9)	12.4 (4.1)		14.5 (2.5)	12.5 (4.2)	
Median	18.5	12.3		14.6	12.3	
Range	12.4-21.6	0-27.6		10.2-18.2	0-27.6	

*t-test for unequal variances used

The cumulative incidence inside the most likely cluster was 17.6 per 100,000 persons (range 12.4-21.6) while outside the cluster the rate was 12.4 per 100,000 persons (range 0-27.6). The cumulative incidence inside the secondary high-risk spatial cluster was 14.5 per 100,000 persons (range 10.2-18.2) (Table 3).

## Discussion

We investigated the spatial epidemiology of suicides in Kentucky as reported in death certificate data files from the Kentucky Office of Vital Statistics between 1999 and 2008 using scan statistics and descriptive epidemiological methods. The results show evidence of hotspots of suicides across Kentucky counties, and describe the differences in suicide characteristics between genders, and for cases inside and outside high-risk spatial clusters. Further, when the purely spatial cluster analysis, SEB map, and cumulative incidence rates are jointly examined, they bolster the evidence of the existence of a high-risk suicide cluster in western Kentucky. To our knowledge this is the first study to investigate spatial and temporal patterns of suicide mortality at the county level in the US. This study allows for a better understanding of where to target resources and prevention efforts at the county level to reduce the burden of suicide in areas of greatest risk [30,31].

Moreover, suicide victim characteristics within the two spatial clusters allow for more targeted prevention efforts. For example, there is a moderately older age group of victims in the most likely cluster, and a greater proportion of 40-44 year old suicide victims inside the secondary cluster compared to outside the cluster. In addition, suicide cases inside the secondary cluster are more likely to self-poison than outside the cluster. Although our results reveal that females are more likely to self-poison than males (and this agrees with national data) [1], there were not significantly more female suicides inside the secondary cluster than outside. These divergent characteristics of suicide cases inside versus outside clusters can inform interventions and guide future studies. Suicide risk was also found to be highest in 2006, providing evidence that suicide has been gradually increasing in Kentucky since 1999.

Our study differs from Exeter and Boyle's (2007) Scotland suicide cluster analysis in several ways [10]. Exeter and Boyle's study, which was limited to young adult suicides (15 to 44 years old), found high risk clusters in Glasgow, Scotland, across three time periods with relative risks ranging from 1.53 to 2.41, while our study revealed clusters with relative risks ranging from 1.24 to 1.38. Unlike our study, where 67% of suicides were firearm related (65% for adults 15 to 44 years old), Exeter and Boyle found that 63% of suicides among young adults were from poisonings, hangings, strangulation and suffocation. In addition, Scotland's suicide rate among adults aged 15 to 44 years in 1999 to 2001 was 24.3 per 100,000 persons, which was markedly higher than Kentucky's suicide rate of 13.5 per 100,000 persons among adults aged 15 to 44 years during the same period [1]. This difference in suicide method and rates suggests that suicide may be better explained by other factors-such as low socioeconomic status [3,10], less access to mental health care [32], sudden unemployment [33], depression [34], and alcoholism [35]-than by firearm access alone [36], given that Scotland's suicide rate is higher than Kentucky's despite there being lower access to firearms in Scotland than Kentucky [37]. Thus, this comparison suggests that firearm availability may not be the sole driving force behind increased regional suicide rates. Both studies reveal that suicide does not occur randomly in space, and that the characteristics of suicide cases inside clusters tend to be different than outside; specifically in Scotland where suicide clusters have been explained by the concentration of socioeconomic deprivation [10].

### Strengths, limitations, and future research

In addition to the previously mentioned advantages of using scan statistics over other epidemiology methods, scan statistics do not assume that observations are spatially independent, but rather test for spatial randomness, or in other words, spatial independence of the observations. An additional strength of this study is that it uses novel spatial techniques to provide ecological information on suicide risk, thus having the potential to guide interventions in those high-risk counties.

This study used data from death certificates which can be affected by errors. Pierce and Denison (2006) assessed place-of-residence errors on death certificates in only two Texas counties and found a 14% error rate in recording county of residence for deaths [38]. Within our dataset, cases where the underlying cause of death and the manner of death did not match were excluded. Further, excluded cases due to this discrepancy came mainly from 2000 and 2001. This differential distribution in excluded cases may have biased the temporal analysis results away from the null hypothesis. Although 2006 has the highest suicide incidence rate, this introduced bias may have inflated that excess risk because of the seemingly lower rates in 2000 and 2001. Additionally, suicide mortality may not reflect current prevention needs. This study also used county-level data, which does not discriminate among suicide mortality risk in different parts of the county.

We are inclined to recommend future studies be undertaken in the high-risk counties to identify reasons for the high rates observed. Although Exeter and Boyle's (2007) study found high-risk suicide clusters to be explained by greater social deprivation [10], implying that greater suicide risk is dependent on a relatively stable regional risk factor, several interventions such as physician education in depression recognition and treatment, along with greater restriction of access to lethal methods have been shown to be effective in reducing suicide rates [39]. It is important to note that since suicide is affected by sociocultural factors, effective interventions in a certain population may not work elsewhere [40]. Regardless, our study does offer evidence to support increasing availability and access to mental health care facilities, and targeted prevention efforts across the identified high-risk clusters in western Kentucky. Given that suicide is highly associated with poor mental health and depression [3,41], it is appropriate to make mental health facilities more available to provide services to those populations that most need it. We believe that future studies assessing suicide risk may provide more insight into regional-specific interventions that are most appropriate for western Kentucky.

In guiding future suicide spatial research, we recommend that individual-level circumstance data from the National Violent Death Reporting System (NVDRS) in Kentucky be linked to socioeconomic and demographic data, and vital statistics data to offer a richer understanding of those persons who define the cluster. It is also recommended that spatial analyses be performed using NVDRS suicide data at the census-tract level to allow comparisons between the county-level analysis and to offer a lower level (i.e., finer) visualization of suicide risk. This study also guides future small area analysis research: i.e., given that two high-risk suicide clusters have been identified, we recommend a spatial analysis limited to each cluster, with those high-risk counties divided into smaller census-tracts to identify those tracts within high-risk counties that are at greatest risk for suicide. Moreover, a further investigation determining the factors associated with high-risk clusters of suicide is recommended using regression analysis by linking various sociodemographic and environmental county (or census-tract) characteristics to the vital statistics data. This approach would offer an ecological understanding of the county-level (or census-tract) characteristics that explain suicide risk. We hypothesize that similar results may be found in Kentucky as in Glasgow, UK, and Queensland, Australia, where socioeconomic deprivation has been associated with high-risk suicide clusters [10,11]. Without more rigorous regression analyses, however, we would only be speculating as to what explains high-risk county clusters of suicide in Kentucky.

Beyond regional social deprivation, several other factors may be contributing to the higher rates of suicide in these relatively rural regions (i.e., all seven counties in the most likely cluster are rural, and three out of eight counties in the secondary cluster are rural) [42]. Specifically, rurality has been found to be a likely regional risk factor for suicide in the United States and Australia [43,44]. Rural populations have less access to mental health care facilities, putting those with mental health disorders at a greater risk for suicide than their urban counterparts [44,45]. Furthermore, data collected from the Kentucky Violent Death Reporting System (KVDRS) from 1999-2008 suggest that county-level unemployment may be statistically associated with higher rates of suicide (Sabrina Walsh, unpublished data).

## Conclusion

This study found high-risk clusters of suicide in western Kentucky and demonstrated the usefulness of the combination and complementary nature of spatial statistics, cumulative incidence, relative risk, and SEB smoothed rates in identifying areas at highest risk for suicide. The findings can guide intervention efforts at the county level in Kentucky to reduce suicide in those areas at greatest risk.

## Abbreviations

US: United States; WHO: World health organization; IRB: Institutional review board; ICD-10: International classification of diseases: 10th revision; CDC: Center's for disease control and prevention; SEB: Spatial empirical Bayesian; SEER: Surveillance: epidemiology: and end results; NVDRS: National violent death reporting system.

## Competing interests

The authors declare that they have no competing interests.

## Authors' contributions

DMS conceived the research idea, study design, performed all analyses, managed data, interpreted results, and wrote the manuscript. AO was involved in study design, results interpretation, and editing of the manuscript. AB drafted the Introduction and was involved in data acquisition. SW was involved in data acquisition, results interpretation, as well as review and editing of the manuscript. All authors certify that they have participated sufficiently in the research to believe in its overall validity and have read and approved the final manuscript.

## Pre-publication history

The pre-publication history for this paper can be accessed here:

http://www.biomedcentral.com/1471-2458/12/108/prepub
